# Case report: Gene mutations and clinical characteristics of four patients with osteopetrosis

**DOI:** 10.3389/fped.2023.1096770

**Published:** 2023-03-14

**Authors:** Yu Chen, Lina Zhou, Xianmin Guan, Xianhao Wen, Jie Yu, Ying Dou

**Affiliations:** ^1^ National Clinical Research Center for Child Health and Disorders, Ministry of Education Key Laboratory of Child Development and Disorders, Chongqing Key Laboratory of Child Infection and Immunity, Children’s Hospital of Chongqing Medical University, Chongqing, China; ^2^ Department of Hematological Oncology, Children’s Hospital of Chongqing Medical University, Chongqing, China

**Keywords:** osteopetrosis, CLCN7, TCIRG1, mutation, variants

## Abstract

Osteopetrosis is characterized by increased bone density caused by decreased osteoclasts or dysfunction of their differentiation and absorption properties, usually caused by biallelic variants of the *TCIRG1(OMIM:604592)*and *CLCN7(OMIM:602727) g*enes. Herein, the clinical, biochemical, and radiological manifestations of osteopetrosis in four Chinese children are described. Whole-exome sequencing identified compound heterozygous variants of the *CLCN7* and *TCIRG1* genes in these patients. In Patient 1, two novel variants were identified in *CLCN7:c.880T > G(p.F294V)* and *c.686C > G(p.S229X)*. Patient 2 harbored previously reported a single gene variant *c.643G > A(p.G215R) in CLCN7*. Patient 3 had a novel variant *c.569A > G(p.N190S)* and a novel frameshift variant *c.1113dupG(p.N372fs)* in *CLCN7*. Patient 4 had a frameshift variant *c.43delA(p.K15fs)* and variant *c.C1360T* in *TCIRG1*, resulting in the formation of a premature termination codon *(p.R454X)*, both of which were reported previously. Our results expand the spectrum of identified genetic variation in osteopetrosis and provide a deeper understanding of the relations between genotype and clinical characteristics of this disorder.

## 1. Introduction

Osteopetrosis encompasses a group of rare metabolic bone diseases characterized by impaired osteoclast activity or development, resulting in high bone mineral density (1). The disease is classified into three major clinical subtypes: autosomal recessive osteopetrosis (ARO), intermediate autosomal osteopetrosis (IAO), and autosomal dominant osteopetrosis (ADO). Patients with ARO, the most severe osteopetrosis type, usually present various fatal manifestations soon after birth and die in infancy or before age three years. Apart from the general manifestations of osteopetrosis, patients with ARO can present with pancytopenia, hepatosplenomegaly, cranial nerve compression, and hydrocephalus (2). The clinical course is often serious and, if left untreated, fatal within the first year of life. Diagnosis is challenging and often delayed or misdiagnosed (1). The spectrum of CLCN7-related osteopetrosis includes infantile malignant CLCN7-related ARO, IAO and ADOII.*CLCN7* (13%–16%) and *TCIRG1* (51%–53%) are the major obligate genes responsible for ARO. *CLCN7* encodes the H(+)/Cl(−) exchange transporter 7, which provides chloride conductance across lysosomes in osteoclasts, ensuring the acidification necessary for cellular function (3). ARO is most often caused by biallelic defects in the gene TCIRG1.*TCIRG1* encodes the a3 subunit, an essential isoform of the vacuolar ATPase proton pump involved in both acidification of the osteoclast resorption lacuna and secretory lysosome trafficking (4, 5). *CLCN7* plays a synergistic role when hydrogen ions are transported outside the cell by *TCIRG1* (6).

## 2. Case descriptions

Herein, we report the clinical features and genetic findings of four patients with osteopetrosis.

Patient 1, a boy aged 2 months and 21 days, presented initially with anemia, skin hemorrhage, and hepatosplenomegaly. His physical examination showed: pale complexion and moderately pale lips, facial skin, and earlobes; neck palpable, ∼5–6 lymph nodes ∼1.0cm × 1.0 cm, axillary and abdominal grooves could be palpated, and several lymph nodes enlarged by ∼0.5cm × 0.5 cm. Laboratory findings showed white blood cell (WBC) 18.04 × 10^9^/L, platelets (PLT) 40 × 10^9^/L, hemoglobin (Hb) 80 g/L, lymphocyte percentage 0.52, neutrophil percentage 0.2, and lactate dehydrogenase (LDH) 1429.6 U/x Imaging examination showed bilateral axillary lymph nodes, bilateral cervical and inguinal lymph node lesions, and spleen enlargement. Bone density was increased, and the medullary cavity was unclear; transverse translucent bands were seen in the metaphysis of the long bones of the limbs, and thin periosteal shadows were seen in the double radius and femur.

Patient 2 was a girl aged 2 years and 3 months who was followed up for bilateral femoral and tibial fractures. Laboratory results showed WBC 6.92 × 10^9^/L, PLT 256 × 10^9^/L, red blood cells (RBC) 3.92 × 10^12^/L, Hb 10^7 ^g/L, lymphocyte percentage 0.315, neutrophil percentage 0.602, and LDH 914.5 U/L. Imaging showed that the cranial cavity was deformed and elongated, the anteroposterior diameter was prolonged, the cranial plate was thickened to varying degrees, fractures of both femurs and tibias, and slightly widened anterior space of the right femoral neck. The bones of both hips, femurs, tibia, fibula, and osseous bones of the foot were obviously dense. The liver and spleen lymph nodes showed no obvious abnormalities.,The patient had no renal complication,and her father hasn't noticed any symptoms about osteosclerosis.

Patient 3 was a boy aged 1 year and 10 months. His head mass was identified at 1year and 8 months, with a hard quality and swelling around the frame and uneven bone density detected. Laboratory examination showed: WBC 8.93 × 109/L, PLT 245 × 109/L, RBC 5.82 × 1,012/L, Hb 112 g/L, lymphocyte percentage 0.3, neutrophil percentage 0.62, and LDH 599.1 U/ L. Imaging showed slightly enlarged bilateral neck and axillary lymph nodes, without obvious liver or spleen abnormalities. The bone density of bilateral ribs, cervical thoracic vertebra, clavicle and right humerus was uneven, scattered in multiple patchy high-density areas, the boundary was not clear, the double lung texture increased and thickened, and the boundary was blurred.

Patient 4, a boy aged 1 years and 15 days, presented at the hospital five times prior to diagnosis. The first four were due to pulmonary infections, from which he was discharged after improvement with antibiotics. At the fifth admission he presented mainly with anemia, thrombocytopenia, hepatosplenomegaly, cough, and shortness of breath. Physical examination showed slightly pale complexion and bilateral conjunctiva, hyperemic throat, and a mild inspiratory three-concave sign. Laboratory examination showed: WBC 14.34 × 10^9^/L, PLT 45 × 10^9^/L, RBC 2.78 × 10^12^/L, Hb 86 g/L, lymphocyte percentage 0.74, neutrophil percentage 0.14, and LDH 617.1 U/L. Imaging showed: slightly enlarged lymph nodes of the liver and spleen; slightly separated left renal pelvis; generally increased density of the craniofacial bones, pelvis, both ends of the ribs, bilateral humerus, ulna, radius, femur, tibia, and fibula. The boundary between the medullary cavity and the cortical bone was unclear, density of the metaphysis was uneven, and different degrees of periosteal reaction could be seen. Radiographs showed bilaterally increased calcaneus and talar bone density. The long bones of the extremities were increased in density, the medullary cavity was unclear, and the metaphysis was difficult to see but slightly widened and thickened, and the lower segment showed translucent shadows. Neurological examination revealed neuromotor retardation,intellectual disability, and right hearing loss. Bone marrow biopsy showed obvious calcification of trabecular bone, intertrabecular fibrosis, and trilineage visible. Some bone marrow hematopoietic cells were significantly squeezed.

### 2.1. Clinical and laboratory findings

**Figure 1:**
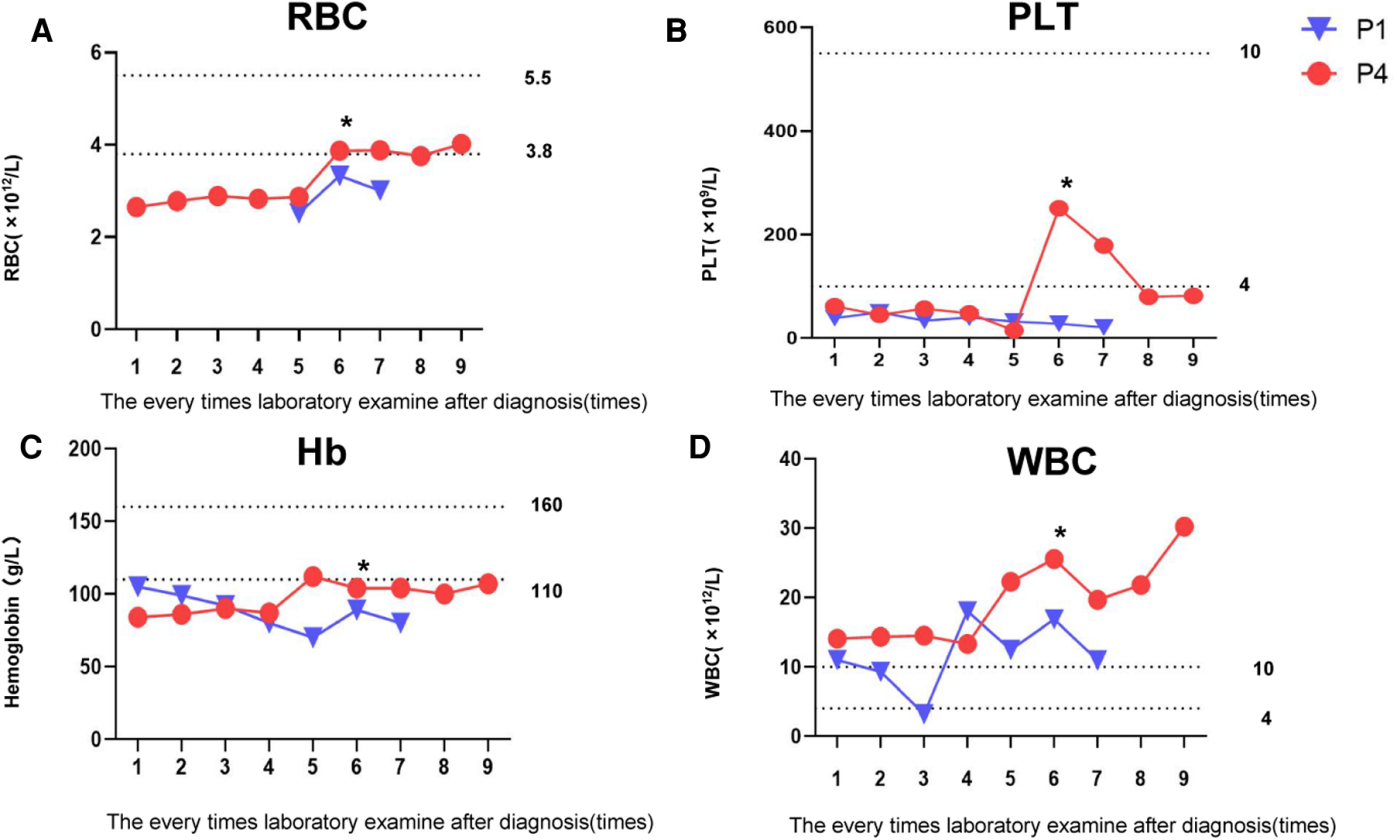
Changes in patients' routine blood examination. (**A**) Changes in patients’ RBC levels. (**B**) Changes in patients' PLT levels. (**C**) Changes in patients' Hb levels. (**D**) Changes in patients' WBC levels. *Transfusion treatments: Patient 1 received RBC 0.25 U; Patient 4 received PLT 0.75 U.

Routine blood tests in Patients 1 and 4 showed that RBC, PLT, and Hb always remained below normal levels (Figures 1A–C). Before the sixth test results, Patients 1and 4 received RBC 0.25 U and PLT 0.75 U transfusion treatments, respectively; each soon thereafter fell below normal levels. These results were consistent with long-term anemia in patients with osteopetrosis. WBC findings in Patient 4 were always higher than normal, possibly associated with recurrent respiratory infections (Figure 1D). Clinical and laboratory data of the four patients are presented in Table 1. Averaged results of multiple assessments, and their standard deviations, were calculated using GraphPad Prism 8.0.

**Table 1:** Summary of clinical findings of the patients.

Patients	P1	P2	P3	P4
**General information**				
Sex	Male	Female	Male	Male
Age at diagnosis	2 months 21 days	2 years 3 months	1 year 10 months	1 month 15 days
**Initial diagnosis results of blood chemistry**				
RBC (3.8–5.5 × 10^12^/L)	2.95 ± 0.41	3.92	5.82	3.28 ± 0.57
WBC(4–10 × 10^9^/L)	11.7 ± 4.94	6.92	8.93	19.5 ± 5.96
Platelets (100–550 × 10^9^/L)	34.86 ± 9.32	256	245	91 ± 75.23
MCV (80–100 fL)	87.33 ± 0.80	89.3	58.9	88.25 ± 6.34
MCH (26–32 pg)	27.03 ± 0.67	27.3	19.2	29.82 ± 4.24
MCHC (320–360 g/L)	309.33 ± 5.51	305	327	339.5 ± 54.27
RDW% (<15.5%)	20.33 ± 0.23	18	16.3	19.92 ± 3.03
lymphocytes absolute value (2–6.5 × 10^9^)	6.4 ± 1.7	2.18	2.68	11.04 ± 3.0
neutrophils absolute value (1.3–6.7 × 10^9^)	3.82 ± 1.12	4.17	5.54	5.15 ± 2.60
Hemoglobin (110–160 g/L)	87.85 ± 12.10	70	112	97.11 ± 10.43
Total bilirubin (1.8–21 umol/L)	33.4	244.9	2.8	19.23 ± 10.10
Direct bilirubin (0–6.7 umol/L)	13.1	244.9	2.8	15.5 ± 11.90
Albumin (38–52 g/L)	37.9	38.3	41.8	39.4 ± 7.34
ALT (0-40 U/L)	58.3	29.1	26.2	38.7 ± 7.64
AST(0-45 U/L)	194.6	64.6	99.4	53.1 ± 14.14
AST/ALT (0.23–2.47)	3.34	2.22	3.79	1.36 ± 0.99
LDH (110–330U/L)	1429.6	914.5	599.1	662.05 ± 63.57
ALP (<500 IU/L)	546.1	119.4	210.7	451.7
Calcium (2.2–3.0 mmol/L)	2.42	1.88	2.52	2.22 ± 0.04
Phosphorus (1.29–2.26 mmol/L)	0.98	2.54	1.84	1.35 ± 0.247
PT	0.89	11.1		12.15 ± 0.50
APTT (9–12.8)	10.7	12.2		29.8 ± 8.06
Fibrinogen (1.1–3.3 g/L)	1.73	2.02		1.30 ± 0.15
TT (19.5–35.4S)	24.7	18.4		19.15 ± 2.33
D-Dimer (<0.55)	9.3	8.92		21.11 ± 3.42
**Other findings**				
Hepatosplenomegaly	Yes	No	No	Yes
Increased bone density	Yes	Yes	Yes	Yes

WBC, white blood cell; RBC, red blood cell; RDW%, Red blood cell volume distribution width; PLT, Platelets; Hb, Hemoglobin; LDH, lactate dehydrogenase; ALP, alkaline phosphatase; PT, Prothrombin Time; APTT, activated partial thromboplastin time; TT, Thrombin time.

### 2.2. X-ray examination and bone marrow biopsy

**Figure 2:**
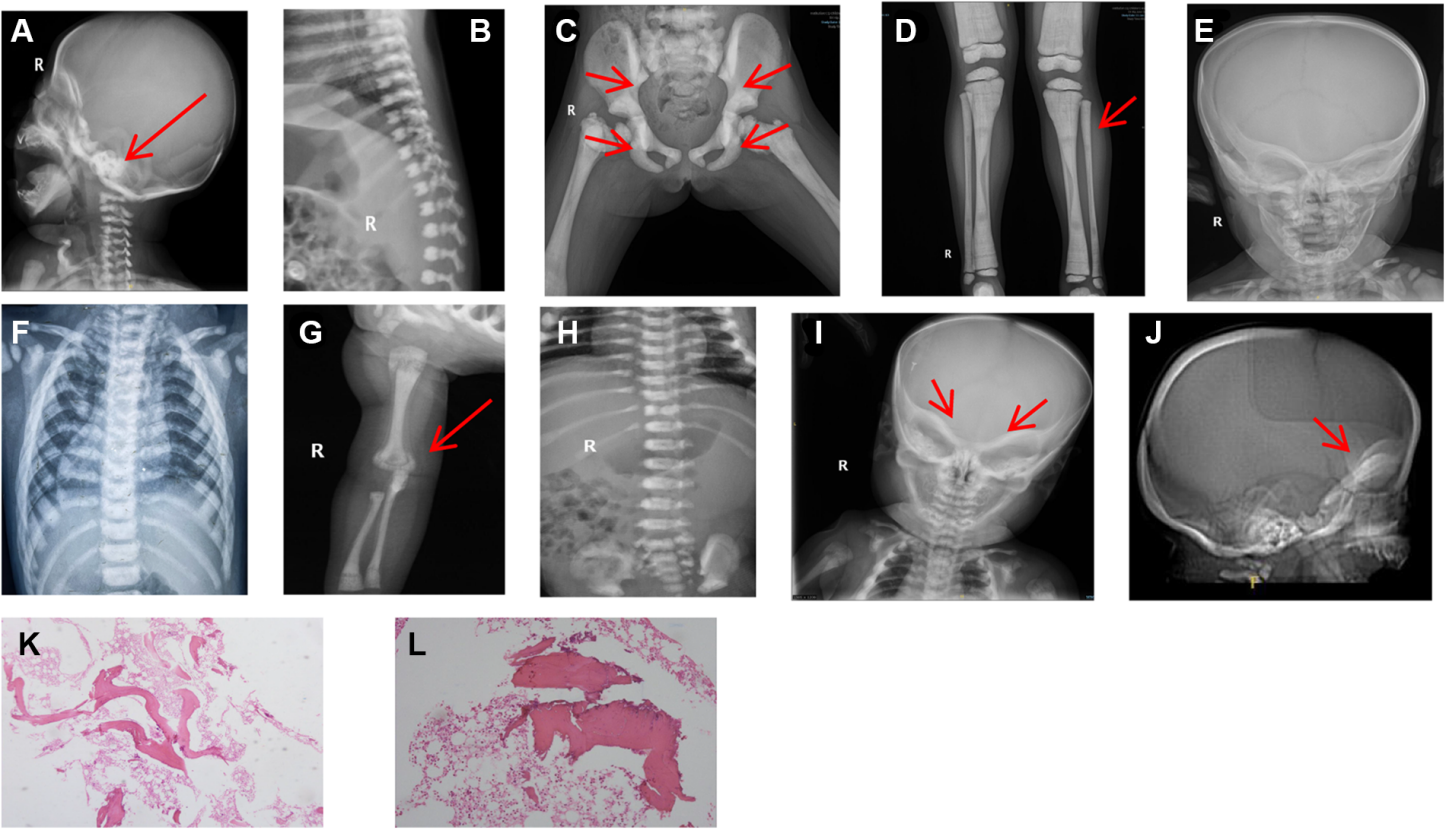
Patient x-rays. (**A**) Patient 1: significant increase in bone density observed at skull base (arrow). (**B**) Patient 1: vertebral endplate showed typical “sandwich vertebrae” appearance. (**C**) Patient 2: generalized increase in bone density and typical pelvic wing “bone-in-bone” evident (arrow); high-bone density under the cartilage in the pelvis (arrow). (**D**) Patient 2: diffusely increased bone density confirmed and osteopetrosis in the distal metaphysis of the femur showed typical “bone-in-bone” appearance (arrow). (**E**) Patient 3: head mass was identified with a hard quality and swelling around the frame and uneven bone density detected. (**F**) Patient 3: vertebral endplate showed typical “sandwich vertebrae” appearance. (**G**) Patient 4: diffuse increase in bone density at the upper radius (arrow), showing “bone-in-bone” appearance. (**H**) Patient 4: vertebral endplate appeared thickened, with “sandwich vertebrae”. (**I**) Patient 4: observed osteopetrosis in skull base (arrow). (**J**) Patient 4: lateral skull computerized tomographyshowing increased thickness of skull base (arrow). (**K-L**) Patient 3: marrow biopsy results showed partial calcification of trabecular bone; HE staining × 40 (**K**); HE staining × 100 (**L**).

The diagnosis of osteopetrosis was also based on the skeletal radiographs.The four patients in our study,imaging examinations consisting of computerized tomography (CT) scans and x-rays revealed a general increase of bone density involving the skull, vertebrae and limbs. And marrow biopsy results in patient3 showed partial calcification of trabecular bone (Figure 2).

### 2.3. Whole-exome sequencing

Genetic analysis is pivotal to the ARO diagnostic work-up. Genomic DNA samples were extracted from peripheral blood using a QIAamp DNA Mini Kit (Qiagen, China) and samples from both the patients and their parents were sent to a third-party testing company (Beijing Hyster Clinical Laboratory). In this method, the target regions of disease-related genes were captured and deeply sequenced with an average sequencing depth of 500–1000x. The overall coverage can reach more than 99%.

The identified variants in *TCIRG1* and *CLCN7* likely caused the manifestations described above.

**Figure 3:**
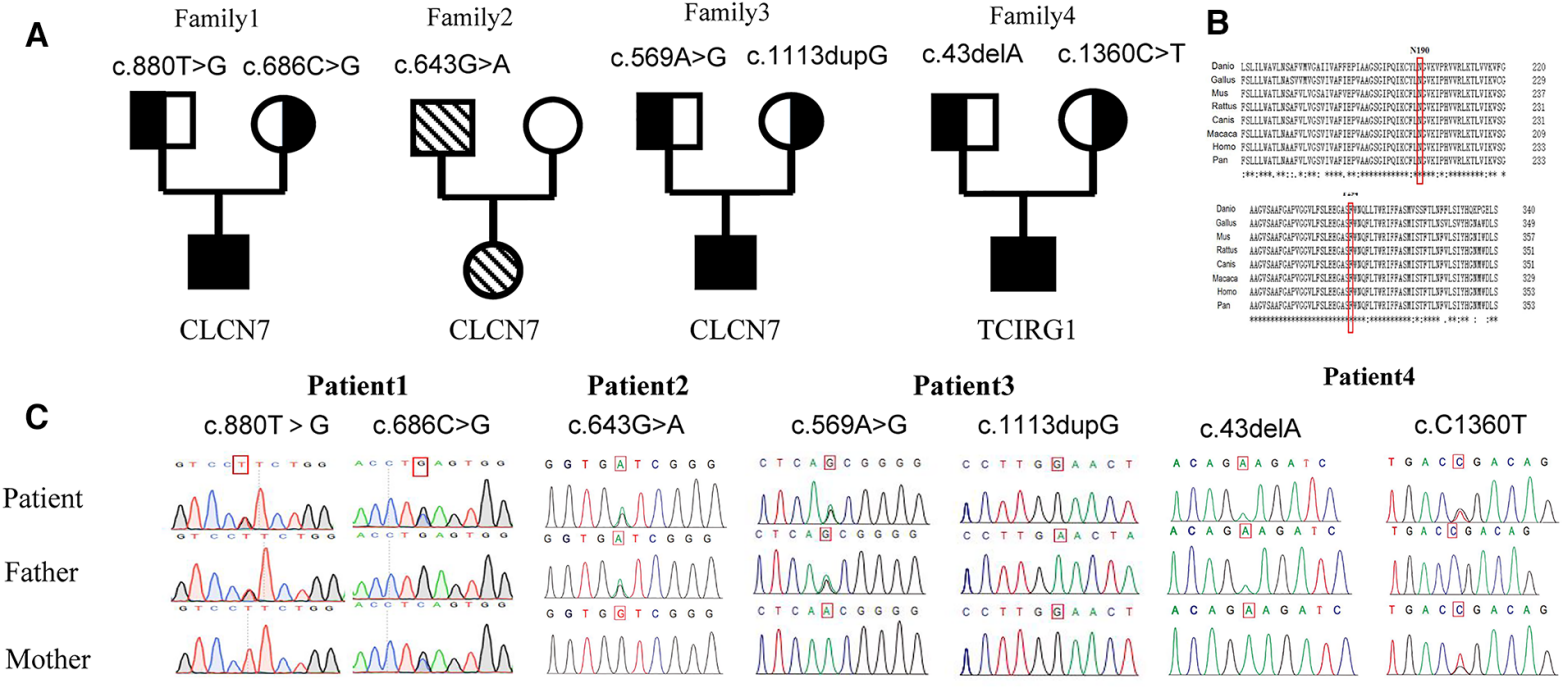
Family pedigrees and genetic sequencings. (**A**) Pedigree for each patient. (**B**) Genetic analysis of the gene mutation in family of Patient 1: compound heterozygous variants c. 880T > C(*p*. F294V) and c. 686C > G(*p*. S229X) in the CLCN7 gene. Homologous sequences of *CLCN7* across species (**C**). A cross-species alignment of amino acid sequences showed that *p*. F294V and *p*. N190S variants occur in a highly conserved region.

In Patient 1, two novel variants were identified in the *CLCN7*(NM_001114331) gene: *c.880T > G(p.F294V)* and *c.686C > G(p.S229X)*. Patient 2 harbored a previously reported hotspot variant *c.643G > A(p.G215R)* in *CLCN7* (7). Patient 3 had a novel variant *c.569A > G(p.N190S)* and a novel frameshift variant *c.1113dupG(p.N372fs)* in *CLCN7*. Patient 4 had a frameshift variant *c.43delA(p.K15Fs)* and a variant *c.C1360T* in *TCIRG1*, resulting in the formation of a premature termination codon *(p.R454X)*, both of which have been previously reported (8). And each patient the variants are inherited from their parents (Figure 3).

### 2.4. Pathogenicity predictions of the *c.880t *>* G(p.F294v)* and *c.569a > G(p.N190s)* variants in the *CLCN7* gene

Potential pathogenicity of the validated missense variant was predicted using SIFT (https://www.siftwallet.com/), PolyPhen-2 (http://genetics.bwh.harvard.edu/pph2/), MutationTaster (https://www.mutationtaster.org/), MutationAssessor (http://mutationassessor.org/r3/), FATHMM (http://fathmm.biocompute.org.uk/inherited.html), PROVEAN (https://www.jcvi.org/research/provean), and CADD (https://cadd.gs.washington.edu/). NCBI gene (https://www.ncbi.nlm.nih.gov/gene) was used to analyze the conservation of the mutated amino acid. gnomAD(https://www.gnomad-sg.org).

The *CLCN7 p.F294V* and *p.N190S* variants, observed in Patients 1 and 3, respectively, occur in the voltage-gated chloride channel domain, which is highly conserved in multiple species. In addition, amino acids at positions 294 and 190 in the *CLCN7* protein are highly conserved.

Next, we evaluated the pathogenicity of the *c.880T > G(p.F294V)* and *c.569A > G(p.N190S)* variants using seven prediction software types. The prediction results for *c.880T > G(p.F294V)* are listed in Table 2, and indicated that this variant is possibly deleterious and implied that it exerts a medium effect on the function of the *CLCN7* protein.And the frequency in general people is 0 which means it's probably pathogenic. The prediction results for *c.569A > G(p.N190S)* are also in Table 2, and suggest that this variant is damaging and show that it exerts a low effect on the function of the *CLCN7* protein.And the frequency in general people is 8.179*10^−6^. These findings indicate that both variants in the *CLCN7* gene are pathogenic.

**Table 2:** Pathogenicity predictions of c. T880G, *p*. F294V and c.569A > G, *p*. N190S in CLCN7 gene.

Prediction software	c.T880G,*p*.F294V		c.569A > G, *p*. N190S	
	Score	Prediction	Score	Prediction
SIFT_score	0.001	Damaging	0	Damaging
Polyphen2	1	Probably_damaging	0.988	Probably_damaging
MutationTaster	1	Disease_causing	1	Disease_causing
MutationAssessor	2.74	Medium (functional impact)	1.47	Low (functional impact)
FATHMM	−3.33	Damaging	−1.75	Damaging
PROVEAN	−6.76	Damaging	−4.83	Damaging
CADD	25.3	Damaging	23.6	Damaging

## 3. Discussion

Herein, we reported on four patients with osteopetrosis, in whom we identified compound heterozygous variants in the *CLCN7* and *TCIRG1* genes. Among the variants, *c.880T > G*, *c.686C > G*, *c.569A > G*, and *c.1113dupG* in *CLCN7* are novel. Two novel missense *CLCN7* variants, *c.880T > G(p.F294V)* and *c.569A > G(p.N190S*), occurred at positions highly conserved across multiple species. In addition, both variants were predicted to be deleterious by multiple in silico tools (Table 2), indicating impaired function of the *CLCN7* protein.

*CLCN7* gene mutations are involved in the pathogenesis of various forms of osteopetrosis. It is located on human chromosome 16p13.3, contains 25 exons, and encodes the 803 amino acid chloride channel protein 7 (CIC-7) (3). Chloride channels are responsible for chloride ion transport and play a key role in the generation and transmission of cellular electrical signals. *CLCN7* is a member of the voltage-gated chloride channel protein family, which mediates the exchange of chloride ions for protons and maintains an acidic environment for bone resorption. *CLCN7* is essential for efficient proton pumping, due to its role in neutralizing currents. It is also involved in the secretion of acid into the resorption void, a specialized acidic compartment for mineral bone matrix degradation. Disruption of *CLCN7* expression results in severe lysosomal storage disorders that, in addition to osteopetrosis, can lead to neurodegeneration, including retinal atrophy (9–11).

The *TCIRG1* gene is located on human chromosome 11q13.2 and contains 22 exons (12), encoding the 830 amino acid a3 subunit of V-ATPase. V-ATPase is a proton pump, the main function of which is pumping hydrogen ions into secretory lysosomes. Osteoclasts degrade bone through acidification by vesicle-like V-ATPase (13). When hydrogen ions are pumped out of osteoclasts, they acidify the cortical environment between osteoclasts and bone tissue, promoting bone resorption and regulating bone formation and development. *CLCN7* cooperates with the gene product of the a3 subunit *TCIRG1* of V-ATPase, acting synergistically when hydrogen ions are transported outside the cell by *TCIRG1* (14, 15).

Besides radiology and bone marrow pathology findings are also very important to the differential diagnosis of osteopetrosis helping to distinguish anemia from leukemia.The main pathological disease change is a defect in osteoclast function, which normally degrade bone to initiate remodeling by maintaining formation of calcification groups. Though many osteoclasts remain, they are dysfunctional and thus during osteogenesis the cartilage matrix continues and the calcified osteoclast cannot break down and absorb normal bone resorption activity which calcification of the cartilage and bone tissue cannot be replaced by normal bone tissue and accumulation that bone tissue cannot rebuild bone density. The hard bones brittle fracture easily and marrow cavities shrink, disappear, or occlude (16).

Three major clinical forms of osteopetrosis have been described,: ADO, autosomal recessive osteopetrosis (ARO), and IAO. ADO is the most common and can be roughly divided into three types: ADOI, ADo-II and ADO-III. The expression of ADOI type was relatively mild, mainly caused by *LRP5* gene mutation. *CLCN7* gene is considered to be the main pathogenic gene causing type II, with penetrance ranging from 60% to 90%. Studies on ADOIII type are still lacking, which is mainly caused by *PLEKHM1* gene mutation. Patients 1 and 4 had severe ARO, onset of which usually occurs in the first year of life and is characterized by generalized increased bone density and heterogeneous symptoms, including hematological and neural defects with diverse severity (17). Laboratory results showed that these two patients also had severe anemia, and although both were treated with blood products, they continued to fall below normal levels shortly thereafter.

According to Whyte's research, elevated serum total LDH and AST can distinguish ADO among the sclerosing bone. However, all four patients herein had elevated AST and LDH of varying severities. Mutations of *CLCN7* and *TCIRG1* both impact osteoclasts so that they fail to properly acidify the surrounding cells and the cortical environment between bone tissues, which affects the extra-skeletal tissues and leads to multiple tissue disorders, and ultimately to elevated AST and LDH. The specific mechanisms remain to be further studied in patients with ADO and mouse models (18, 19). At present, genetic diagnosis is still the most important method for confirming osteopetrosis classification.

Patient 2 was diagnosed with ADOII, the main characteristics of this type which are diffuse, including symmetrical osteopetrosis mainly affecting the spine, pelvis, and skull. Severe symptoms include fracture, osteomyelitis, vision loss, and bone marrow failure (20). Among these clinical symptoms, fracture, especially of the long bones, is the most likely independent complication of ADOII. ADOII is an autosomal dominant disorder with incomplete penetrance. The penetrance rate has previously been reported to be between 75% and 94%.There are also asymptomatic carriers of the *G215R* mutation and nonpenetrance rates were 24%. So far, the father of patient 2 has not found any symptoms related to stone bone disease, so he may be an asymptomatic carrier. Almost every adult with ADO (98%) reports fracture. In children with ADO, fractures are less prevalent but still occur in 53% (21). These symptoms were present in Patient 2, who had fractures of both femoral necks. This may be due to impairment of bone remodeling from defective osteoclast function, unsuccessful repair of microdamage, and abnormal bone biomechanics (22). Thus, patients with ADO should be appropriately counseled regarding their propensity for fractures and other ADO-related problems.

Childhood-onset IAO can have autosomal dominant or autosomal recessive inheritance. Its clinical manifestations are intermediate between those of ADO and ARO.The onset of symptoms is usually before the age of first 2 years. Children may present with fractures after minor trauma or characteristic changes on x-rays obtained for other clinical indications. Hematologic signs are milder than those in ARO and are usually restricted to anemia. Inheritance is AD or AR (23). In terms of clinical symptoms, Patient 3 may have this type. Although the clinical symptoms of ADO and IAO are less severe than those of ARO, some patients have been diagnosed with significant clinical manifestations in infancy, which should receive appropriate clinical attention.

Currently, to improve patient survival and quality of life, hematopoietic stem cell transplantation (HSCT) is the only curative treatment for osteopathy. HSCT does not improve the course of osteopetrosis in individuals with *TNFSFII* gene mutation, nor can it reverse the renal tubular acidosis and renal injury caused by *CAII* gene mutation. However, for individuals with *TCIRG1* gene mutation, the effects of transplantation are clearer (1, 24–27). Therefore, genetic testing has guiding significance for whether children can be cured by transplantation. When successful, bone marrow biopsy shows normal results ∼13 weeks post-HSCT. In a mouse model of *TCIRG1* defect, bone performance is partly correct on x-ray and histopathological analysis 8 weeks post-HSCT, and after 18 weeks is almost entirely normalized.

To date, Patient 2 has survived without specific treatment, but remains at risk for fractures. Patients 1 and 3 were lost to follow-up. Only Patient 4 met the conditions for HSCT, but his parents were not satisfied with the match result (HLA8/10) and refused treatment. Treatment efficacy depends on patient genotype. Individuals with *OSTM1* gene abnormalities and some *CLCN7* gene mutations associated with neurodegeneration do not show improvement with HSCT (28).

In summary, four children with osteopetrosis were classified based on clinical manifestations, biochemical examinations, radiological changes, and genetic defects. Accurate osteopathy classification is essential to diagnosis and differentiation from other diseases, as well as to subsequent treatment. Herein we identified novel variants, expanding spectrum of the *TCRIG1* and *CLCN7* genes and deepening our understanding of the relations between genotype and clinical characteristics of osteopetrosis.

## Data Availability

The datasets presented in this article are not readily available because of ethical and privacy restrictions. Requests to access the datasets should be directed to the corresponding author/s.
